# Misinformation Through the Lens of Dutch News Media and Public Health Authorities: Content Analysis of Topics and Narratives Present in Pandemic-Related Misinformation During COVID-19

**DOI:** 10.2196/91743

**Published:** 2026-09-04

**Authors:** David Blanco-Herrero, Bas van den Putte, Toni G L A van der Meer

**Affiliations:** 1 Department of Communication Science, Amsterdam School of Communication Research (ASCoR), University of Amsterdam, Nieuwe Achtergracht 166, Amsterdam, North Holland, The Netherlands, +31 639348327

**Keywords:** COVID-19 pandemic, public health misinformation, vaccines, content analysis, agenda-setting

## Abstract

**Background:**

One of the multiple challenges associated with misinformation is its risk of hindering public health efforts during a health crisis. Misinformation can cover a diversity of thematic topics and is usually spread using a series of nontopic-specific recurring narrative structures. Identifying these 2 misinformation features helps to understand underlying structures and patterns through which misleading information can take shape or be circulated.

**Objective:**

The goal of the study is to obtain a complete overview of the topics and narratives of pandemic-related misinformation cases addressed by 2 key information providers during the COVID-19 pandemic in the Netherlands: news media and public health authorities.

**Methods:**

A quantitative content analysis was conducted on 698 news articles and 1333 tweets published between January 2020 and May 2023.

**Results:**

The study revealed that vaccine-related misinformation, both in terms of topics and narratives, was the most frequently addressed type of misinformation across both sources. The topics and narratives evolved along the pandemic, with the virus origin and danger being more predominant in the misinformation addressed in the early phases and the vaccines gaining presence around 2021.

**Conclusions:**

In total, the study revealed the dominance of vaccine-related narratives as well as the change of misinformation features over time. The deeper understanding of misinformation topics and narratives can inform communication strategies to counter its effects, focusing on the appropriate topics and narrative structures in different moments.

## Introduction

### Background

In a health crisis, misinformation poses a threat that increases the danger of the health emergency. During the COVID-19 pandemic, misinformation affected individual responses to the crisis [1-3], which hindered the efforts to counter the outbreak. Studies reviewing social media content showed that misinformation about vaccine safety and effectiveness erodes confidence in vaccines and decreases intentions to be vaccinated [4,5]. For example, variation in level of misinformation between US states predicted a decrease of 20% vaccine uptake between states [6]. Beyond vaccination, belief in COVID-19 misinformation is associated with reduced adherence to preventive behaviors such as mask-wearing, physical distancing, and hand hygiene, particularly among individuals with lower trust in institutions [7]. More broadly, widespread misinformation about the virus, vaccines, and public health measures was also observed to undermine trust in authorities and support for evidence-based public health policies [8].

To fully understand the implications of misinformation during this period, it is necessary to go beyond assessing its accuracy or effects and also describe its content. For instance, in their report for the European Parliament, Jacob et al [9] identified key COVID-19 misinformation themes in European countries, such as vaccination and the disease severity. Although identifying misinformation topics is a common approach due to its importance for agenda-setting [10], the study of specific narratives or conspiracy theories that articulate these topics are less frequent (though not completely absent; see, for instance, Kotseva et al [11]).

Misinformation topics are conceptualized as broad and neutral issues, while narratives are specific storyline structures. Topics reflect content areas around which misinformation can circulate or not and can be neutral (eg, vaccines), while narratives follow recurring storyline structures that frame elements of one or more topics in a particular direction (eg, vaccines were intentionally created to harm people). Thus, topics focus on *what* the misinformation is about, whereas narratives are the stories people construct around those topics, showing *how* events are connected and *why* the topic matters to an audience. Using both concepts allows for a more complete understanding of misinformation content, capturing not only the main topics but also the more specific recurring narratives used to spread misinformation, a dimension that has received less attention than the study of topics in previous literature.

To obtain a broad view of misinformation in a given context, it is also important to include more than one type of source of information, unlike most prior studies. Because news media shape public opinion and set the public agenda [12], examining their coverage of misinformation remains relevant, as seen, for instance, in Lwin et al [13] regarding COVID-19 misinformation in Singapore. However, other information providers also play a role. Especially during a health crisis, public health authorities are central in countering the effects of misinformation, as they have direct channels to reach citizens [14,15]. Besides their mediated presence in news media or press conferences, their social media accounts serve as more direct communication channels.

A study on the use of Twitter by public health agencies during the early stages of the pandemic [16] found limited attention to correcting misinformation, perhaps because only the first months were analyzed, when misinformation was insufficiently understood and not enough was known about how to correct it. The authors recommended these organizations “strengthen their efforts to combat misinformation and providing more insights into research.” In a similar line, Rasmusen et al [17] found that interventions from public health authorities effectively mitigated the spread of misinformation on social media. Thus, even if addressing misinformation is not always a central target of their communication strategies, public health authorities are well positioned to do so, especially considering that they were the most commonly chosen source by social media users seeking information during the pandemic [18]. However, analyses of their specific approach to misinformation remain scarce.

Therefore, this study introduced 2 main contributions: First, it complemented the analysis of misinformation topics with the analysis of recurrent narratives, and second, it combined 2 types of information sources that addressed pandemic-related misinformation, namely online and print news media and tweets from public health organizations, increasing the diversity of observable strategies and consequently the study’s external validity. Although other media types or social media platforms could have been examined, the predominantly textual nature of these 2 sources allows for a coherent analysis strategy. Additionally, the study covered the full duration of the pandemic, offering a broader view of the evolution of misinformation features than previous studies with narrower time frames.

In total, the main goal of our study was to identify the topics and narratives of pandemic-related misinformation cases addressed by news media and public health authorities in the Netherlands during the COVID-19 pandemic. The objective was not to analyze all the misinformation circulating this period but rather the misinformation considered most important by 2 relevant and diverse sources. Because it is unfeasible to accurately capture all false claims and rumors spreading across formats and platforms, analyzing misinformation addressed by relevant sources offers a valid approach, as seen in the studies by Brennen et al [19] and Ceron et al [20], who focused on the content debunked by fact-checking organizations. These approaches not only illustrate the main topic and structure features of misinformation but also reveal the preferences of the studied sources.

This way, we went beyond previous literature that largely sought to map the themes and narratives circulating among the general public, particularly on social media (eg, [11]), with comparatively less attention to how institutional actors responded to misinformation. By examining the topics and narrative structures of misinformation as they were addressed in journalistic coverage and in the communication of health agencies, we shifted the focus from the spread of misinformation to which misinformation is publicly contested and countered. This perspective foregrounds the agenda-setting and framing role of corrective communication and helps identify which misinformation themes receive attention and when.

In our study, we did not attempt to establish what qualifies as misinformation but rather understand what news media and public health authorities perceive as such. Thus, misinformation was conceptualized as any claim that the studied news articles or tweets addressed as such, without further exploring whether the alleged misinformation cases addressed by the studied actors actually entailed misinformation as defined by scientific definitions (ie, a verifiably inaccurate or misleading assertion about COVID-19, its prevention, or vaccination that conflicted with the best available scientific evidence or official guidance at the time [21]) or whether that also includes normative statements (eg, whether measures were “too strict” or “unfair”) and disagreements over policy preferences without a falsifiable factual component.

Before delving more specifically into the literature that underpinned this research, it is relevant to briefly contextualize the Dutch case, where the study was conducted. In the Netherlands, the government response to the COVID-19 pandemic combined voluntary compliance, personal responsibility, and fluctuating proportional restrictions [22,23]. Throughout the pandemic, Prime Minister Mark Rutte and successive health ministers held regular televised press conferences to explain measures and justify policy choices, with the National Institute for Public Health and the Environment (RIVM) providing weekly epidemiological updates and guidance. The Dutch media environment during COVID-19 was characterized by high consumption by Dutch citizens of traditional news outlets and generally high, although fluctuating, levels of trust in journalism and governmental information [24,25]. There was, however, space for contestation and skepticism and the spread of conspiracy narratives, especially on social media [26], something that even fostered social movements [27]. Thus, the Netherlands combined a relatively trusted, expert-oriented news system, in which the institutional response to the crisis coexisted with contestation that was partly fueled by misinformation and conspiracy theories, making it a relevant case to examine how mainstream news media and public health authorities addressed pandemic-related misinformation.

### Misinformation Topics and Recurring Narratives During the COVID-19 Pandemic

Following the longstanding line of work on agenda-setting [12] and considering as sources both news media and public health authorities, it was relevant to identify the topics that were more predominantly present in misinformation instances, as false information also has the capacity to set the agenda [28]. Previous examples are a report by Bruns et al [29], whose review of empirical evidence from social and behavioral sciences showed that COVID-19 misinformation did not focus only on health aspects but also included misinformation about political responses to the pandemic or the origins of the virus and its severity. Focusing exclusively on the first months of the crisis and using the misinformation corrected by fact-checkers as a reference, Brennen et al [19] and Salaverría et al [30] also studied the main topics present in misinformation cases. Building upon these studies, our study combined a timeframe covering the whole duration of the declaration of pandemic by the World Health Organization (WHO) while also including 2 diverse sources. We asked 3 research questions (RQs).

RQ1 asked “What were the main topics present in the pandemic-related misinformation addressed by printed and online news media as well as Twitter accounts of public health authorities in the Netherlands during the COVID-19 pandemic?”

As an important novelty, this study went beyond topics, trying to also identify misinformation narratives. Topics are the *subject matter* or domain around which misinformation clusters, although they could also be used to structure types of content other than misinformation (eg, vaccination can be a topic around which misinformation spreads but also a broader topic of health information). Narratives, by contrast, go beyond topical labels and relate to recurring storylines and argumentative logics that give meaning and structure to misinformation, with ideas being linked causally and temporally, not just thematically (eg, vaccines are created by an elite to cause harm). Although narratives can differ in their level of complexity, they often include a “cause-and-effect relationships between events that take place over a particular period that impact particular characters” [31].

This narrative dimension plays a distinctive role in the dynamics of misinformation, as it allows rhetorical strategies or constructions that go beyond merely misstated facts. In fact, misinformation has been frequently observed to repeat itself or evolve around specific stories and tropes [32]. Part of the successful spread of misinformation is explained by its capacity to exploit recurring narratives, making use of familiarity biases and the illusory truth effect, which is the tendency to believe that (false) information is correct after repeated exposure [33,34].

The study of misinformation narratives during the COVID-19 pandemic has been tackled in a few studies. For example, Kotseva et al [11] identified 12 super-narratives that were repeatedly observed: Vaccine-related narratives, Criticism restrictions, Conspiracy theories, Criticism of EU and inter/national actors, Claims of authoritarianism and dystopia, Geopolitics, Health-related narratives, Fearmongering, Downplaying COVID-19, Anti-minority narratives, Distrust toward media, and Other. Although with a broader operationalization than the one adopted in our study and collected exclusively on news articles, these super-narratives offer one of the most comprehensive perspectives to date to understand misinformation beyond its topics; they were therefore used to inform our research design and instruments.

However, the investigations addressing misinformation narratives frequently used shorter timeframes that did not cover the whole duration of the pandemic and did not include a diversity of sources for the analysis, especially by looking at the content of public health authorities. To provide a broader overview, we posed the following question (RQ2): “What were the main recurrent narratives present in the pandemic-related misinformation addressed by printed and online news media as well as Twitter accounts of public health authorities in the Netherlands during the COVID-19 pandemic?”

Misinformation topics and narratives were not static. Both Jacob et al [9] and Kotseva et al [11] observed that the presence of misinformation topics and specific narratives changed over time during the pandemic. Further comprehending the time evolution of misinformation features is relevant to understand how they might have been affected not only by the volume of misinformation circulating at a given time but also by additional factors, such as the roles of the sources or the shifting societal goals during the pandemic [35]. Once again, previous research is limited in its partial covering of the pandemic’s duration, and analyses often do not combine the study of diverse misinformation features (ie, topics and narratives) with diverse sources (ie, news media and public health authorities). Therefore, with the goal of studying how the topics and narratives present in the misinformation addressed by news media and public health authorities changed over the duration of the pandemic, we posed the last research question (RQ3): “How did the main (1) topics and (2) recurrent narratives present in pandemic-related misinformation addressed by printed and online news media as well as Twitter accounts of public health authorities in the Netherlands change along the COVID-19 pandemic?”

## Methods

### Procedure

This research was based on a quantitative content analysis of national news media and Twitter posts of public health authorities in the Netherlands. Although the COVID-19 pandemic and the spread of misinformation were global phenomena, the analysis of misinformation features from a national perspective is justified by the possibility of using comparable sources for the analysis as well as by the response to the health crisis, which was mostly determined by national governments and differed from country to country.

The period of analysis comprised from January 1, 2020 (because on January 24, 2020, the first news items about COVID-19 were published in the Netherlands), to May 5, 2023 (day on which the WHO officially ended the pandemic declaration). Given the different phases within the pandemic and in order to answer RQ3, we followed the process by Solovei et al [24] to determine the phases of the COVID-19 pandemic in the Netherlands. These phases were defined based on the waves of infections and hospitalizations [23] and the stringency index developed by Hale et al [36] to reflect the response measures enacted by governments during the pandemic. The 6 phases used in this study were (1) the dawn of the pandemic and the first wave (January 2020-May 2020), (2) the first interwave period of relief (June 2020-August 2020), (3) the second and third waves (September 2020-December 2020 and March 2021-April 2021) with no relaxation of measures in between, (4) the second period of relief (May 2021-September 2021), (5) the fourth wave (October 2021-December 2021), and (6) the final relief period until the end of the pandemic declaration by the WHO (January 2022-May 2023).

Content was collected from national newspapers (both printed and online) and from the Twitter accounts of 5 regional and national public health organizations. Although Twitter was not the most used social media platform in the Netherlands during the period of study—around 20% of the Dutch population were Twitter users according to data from the National Social Media Study [37,38]—the choice of this platform for the analysis is explained by its open and accessible nature as well as by its role as a central stage for public debate and the provision of information by public institutions [39], particularly during the COVID-19 health crisis [40]. This approach was also used by previous studies addressing Twitter communication of public health authorities during the pandemic [41]. It should be noted that the Twitter platform changed its name to X in July 2023; the original name has been consistently used across the manuscript, since that was the name of the platform during the entirety of the studied period.

Although data collection and analysis of news media and Twitter occurred in parallel, the process, materials, and instruments were channel-specific and therefore slightly different for both types of data. In both cases, the strategy was driven by the communication patterns of the sources: Given the greater amount of information, search strings were used for news media, something that was not necessary for the Twitter posts, which could be filtered manually. Regarding the news media, Nexis Uni was used to identify articles dealing with pandemic-related misinformation in the 8 main Dutch national newspapers. Given the limited presence of online-only news media in this database, a complementary search was conducted on Google News for the 2 main digital news platforms in the Netherlands: NU.nl and NOS.nl.

The search string used in these cases was [(nepnieuws* OR misinformatie* OR desinformatie* OR fake OR “valse informatie”) AND (covid* OR corona*)]. This translates into English as [(“fake news” OR misinformation OR disinformation OR fake OR “false information”) AND (covid OR corona)]. The use of wildcards is explained by the possibility of creating words in Dutch by combining terms, such as “desinformatiecampagne,” meaning “disinformation campaign.“ This reflects the study’s focus on news articles in which misinformation was explicitly constructed as such and not necessarily those in which false claims or COVID-19 were discussed implicitly. This search led to 2151 cases, which were subject to a preliminary analysis to identify cases in which pandemic-related misinformation was sufficiently present. This additional screening ensured that only articles where pandemic-related misinformation was a relevant part of the story were retained, removing cases where it was mentioned only as background information (eg, political misinformation during the 2020 US presidential elections where the pandemic was merely contextual). The final sample comprised 698 cases in which pandemic-related misinformation was central to the story. All cases were in Dutch and included news and opinion articles, excluding other formats such as quizzes and weather forecasts.

Regarding the Twitter posts of public health authorities, 5 institutions were selected: GGD (Gemeentelijke Gezondheidsdienst) Amsterdam, GGD Rotterdam-Rijnmond, GGD Utrecht Regio, the RIVM, and the Ministry of Health. The first 3 are regional or local institutions in charge of the implementation of emergency measures during the pandemic: Those were the organizations that led the testing and vaccination process in their region and produced much of the communication that reached their local communities. The 3 selected accounts are those with the largest number of followers and publications. The last 2 are the national institutions in charge of the design of health measures and strategies (eg, vaccination, testing) that the different GGDs would implement. Our selection combines the communication of larger national organizations with the communication of more local and hands-on ones.

For a Twitter post to be selected, it was not enough to provide information on a pandemic-related topic (eg, where the vaccination centers are); it had to convey information that would counter false rumors or help clarify doubts or fears among the population. An example can be found in the post from the Health Ministry on January 19, 2022 (Original Dutch text: “Vragg jij je ook af of je niet beter kunt wachten op een vaccin tegen de omikronvariant? Immunoloog Marjolein van Egmond van het @amsterdamumc, raad dat niet aan. Ze legt uit waarom.”): “Are you also wondering if it wouldn’t be better to wait for a vaccine against the omicron variant? Immunologist Marjolein van Egmond of the @amsterdamumc advises against it. She explains why.” This was followed by a video of this immunologist, in which those concerns were disputed. Although the post did not directly mention misinformation, it alludes to and tries to counter fears and doubts that can be supported by misinformation.

Two trained coders independently analyzed the timeline of each of the 5 accounts during the studied period, searching for posts tackling pandemic-related misinformation or providing information that could be used to counter misinformation. An exploratory observation of the activity of the public health authorities on their social media showed that they do not usually correct misinformation explicitly, but they rather provide information that can be used to counter the effects of misinformation. This led us to adopt a wider conceptualization of the ways of dealing with misinformation, rather than limiting the search to explicit mentions to misinformation. To safeguard reliability, relevant cases were selected only when both coders agreed on their adequacy for the goals of the study, thus ensuring that the selected cases were indeed referring to misinformation. This led to a total of 1333 cases.

Content from both news media and Twitter accounts was analyzed using the same codebook, which was slightly adapted to fit the particulars of both types of content (see Multimedia Appendices 1 and 2). The analysis was preceded by a training process as well as a series of intercoder reliability (ICR) tests to ensure the reliability of the coding. For the news articles and Twitter posts, 95 of the 698 analyzed cases (13.6%) and 135 of the 1333 analyzed cases (10.1%), respectively, were double-coded. The results of these tests are shown in Table 1. Because all the variables were dichotomous and many were highly skewed, we used prevalence-adjusted and bias-adjusted kappa (PABAK) as our primary reliability coefficient, which is less sensitive to prevalence and bias in such situations [42]. Although Krippendorff alpha is widely regarded as a standard reliability index in content analysis, particularly for multicategory nominal data and scale construction [43], given our data structure, the use of Krippendorff alpha was expected to underestimate agreement for rare categories. Therefore, PABAK provided a more informative summary of coder agreement. On average, the ICR values reached a satisfactory 0.89, with only 2 values offering less than substantial (<0.60) agreement; in these 2 cases, caution in the interpretation of the results is recommended.

**Table 1.** Intercoder reliability measures for each variable.

Variable	News outlets, PABAK^a^	Public health authorities, PABAK
Topic (Presence)		
Misinformation on the origin of the virus	0.90	1.00
Misinformation on the danger and spread of the virus	0.80	0.66
Misinformation on vaccines	0.85	0.88
Misinformation on pharmaceutical measures, other than vaccines	0.90	0.69
Misinformation on nonpharmaceutical measures	0.77	0.87
Misinformation on political issues related to the pandemic	0.87	1.00
Misinformation on economic issues related to the pandemic	0.98	1.00
Misinformation on social issues related to the pandemic	1.00	0.90
Misinformation on technological issues related to the pandemic	0.90	0.99
Misinformation on other topics related to the pandemic	0.98	0.97
Narrative (Presence)		
The virus has been intentionally created	0.71	1.00
The virus does not exist or is less harmful than pretended	0.75	0.67
Lockdowns or other measures are used to control the population or to limit their freedom	0.90	0.97
Useless or dangerous remedies are effective	0.96	0.99
Vaccines do not work or are not sufficiently tested	0.94	0.56
Vaccines are intentionally harmful or evil	0.92	0.97
The pandemic is part of larger scheme or conspiracy theory	0.83	1.00
Bill Gates is involved in some way with the virus, the measures, or the vaccines	1.00	1.00
George Soros is involved in some way with the virus, the measures, or the vaccines	1.00	1.00
Other recurring narratives	1.00	0.32

a PABAK: prevalence-adjusted and bias-adjusted kappa.

### Variables

The codebook started with a series of descriptives, followed by the main variables related to the features of misinformation. The descriptive variables included the date of publication, the source, and, for the Twitter posts, the type (original or a retweet). For the study of misinformation topics and narratives, a hybrid deductive-inductive coding strategy was adopted. Following previous studies [9,11,19,29] as well as a set of interviews with information intermediaries who had tackled misinformation during the COVID-19 pandemic in the Netherlands [44], 9 topics and 9 narratives were coded. As a complement, open categories were included to capture additional topics and narratives. The final variables were topics and narratives.

For topics, the presence (1) or absence (0) of the following pandemic-related misinformation topics were coded (more than one option could be selected): Origin of the virus; Danger and spread of the virus; Vaccines; Pharmaceutical measures, other than vaccines; Nonpharmaceutical measures; Political issues related to the pandemic; Economic issues related to the pandemic; Social issues related to the pandemic; Technological issues related to the pandemic; and Other topics (including a follow-up open question).

For narratives, the presence (1) or absence (0) of the following pandemic-related narratives were coded (more than one option could be selected): The virus has been intentionally created; The virus does not exist or is less harmful than pretended; Lockdowns or other measures are used to control the population or to limit their freedom; Useless or dangerous remedies are effective; Vaccines do not work or are not sufficiently tested; Vaccines are intentionally harmful or evil; The pandemic is part of a larger scheme or conspiracy; Bill Gates is involved in some way with the virus, the measures, or the vaccines; George Soros is involved in some way with the virus, the measures or the vaccines; and Others (including a follow-up open question).

### Ethical Considerations

This research was conducted after preregistration [45] and ethical approval from the Ethics Review Board of the Faculty of Social and Behavioral Sciences of the University of Amsterdam (number FMG-9528).

## Results

### Sample Description

The final sample included 698 news articles and 1333 Twitter posts from public health authorities that had dealt with misinformation during the COVID-19 pandemic. The subsample of news articles was dominated by printed newspaper articles (650/698, 93.1%), with only 48 articles from online only newspapers (48/698, 6.9%). The printed newspaper *De Volkskrant* had the most articles in the sample (144/698, 20.6%), and the online only NU.nl had the smallest presence (18/698, 2.6%). Articles were on average 892.15 (SD 765.43) words long, with the shortest article containing 34 words and the longest containing 7318 words.

For the Twitter posts, the 1333 cases were distributed more unevenly, with a clear predominance of GGD in the Utrecht region (793/1333, 59.5%). Given its predominance in the sample, we tested whether GGD Utrecht’s posts showed specific patterns that could affect the overall interpretations, but no relevant differences were observed. In contrast, GGD Amsterdam had only 43 (43/1333, 3.2%) posts in the sample. Most of the posts were original publications (1226/1333, 92%), while the others (107/1333, 8%) were retweets or mentions of existing posts, frequently from the same account or another public health institution.

**Figure 1.**
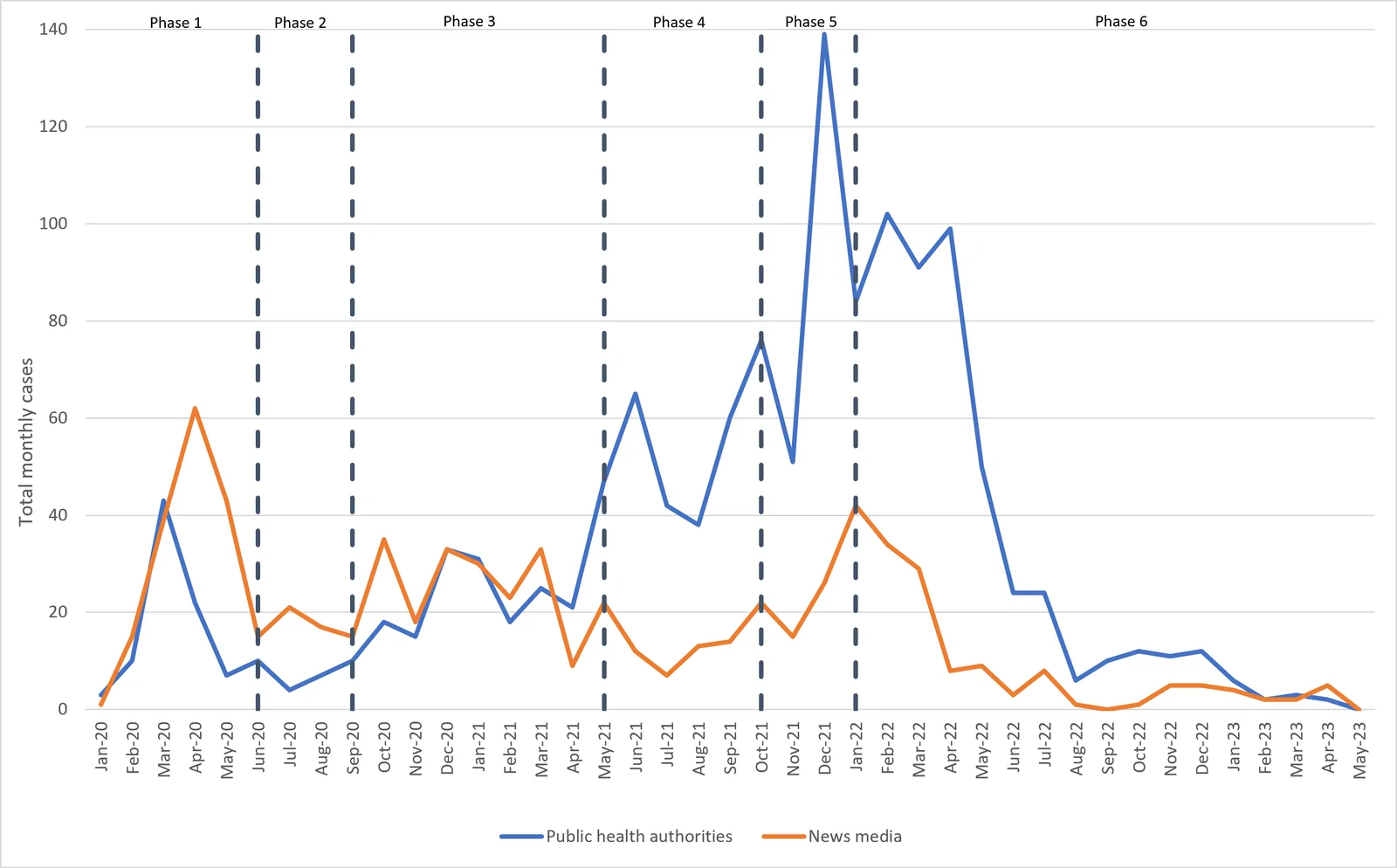
Time distribution of the analyzed cases.

Figure 1 shows the monthly distribution of the number of cases per dataset during the period analyzed. Although some shared patterns can be identified, the peaks in each of the 2 subsamples took place in different moments. The highest number of news articles was published during the first phase, when the first wave of the pandemic surprised the Netherlands and other Western countries. For the public health authorities, the most active period included the months at the end of year 2021 and the first months of 2022, during which the Netherlands experienced an important increase in the number of infections while the vaccination campaign was also ongoing.

### Topics and Narratives

To reply to RQ1, vaccination was the most frequent misinformation topic among both the news articles and the Twitter posts (Table 2). The topics were more diverse in the news articles; besides vaccination, the most frequent topics related to the spread, danger, and origin of the virus and to pharmaceutical measures other than vaccines. In the Twitter accounts of public health authorities, more than one-half (817/1333, 61.3%) of the posts concentrated on vaccination, followed by pharmaceutical and nonpharmaceutical measures to counter the pandemic.

**Table 2.** Presence of misinformation topics in the sample.

Topics	News articles (n=698), n (%)		Twitter posts (n=1333), n (%)	
Misinformation on vaccines (eg, vaccines include microchips)	222 (31.8)		817 (61.3)	
Misinformation on the danger and spread of the virus (eg, the virus is not real)	146 (20.9)		111 (8.3)	
Misinformation on the origin of the virus (eg, the virus was intentionally created)	141 (20.2)		1 (0.1)	
Misinformation on pharmaceutical measures, other than vaccines (eg, hydroxychloroquine kills the virus)	131 (18.8)		342 (25.7)	
Misinformation on technological issues related to the pandemic (eg, the COVID-19 app is used to track citizens)	47 (6.7)		25 (1.9)	
Misinformation on nonpharmaceutical measures (eg, the lockdown is used to control the population)	40 (5.7)		130 (9.8)	
Misinformation on social issues related to the pandemic (eg, immigrants are not respecting the restrictions)	17 (2.4)		13 (1)	
Misinformation on political issues related to the pandemic (eg, alleged declarations of politicians)	16 (2.3)		1 (0.1)	
Misinformation on economic issues related to the pandemic (eg, the pandemic is destroying a particular economic field)	3 (0.4)		0 (0)	
Other topics	0 (0)		0 (0)	
No topic specified	205 (29.4)		126 (9.5)	

In a significant number of cases, no specific topic was identified, especially in the news articles: This was usually the case with messages that addressed false information in the context of the COVID-19 pandemic but did so as a general threat or trying to warn or educate people on the phenomenon of misinformation without connecting it to a specific topic.

Regarding the more specific narratives explored in RQ2, different patterns were observed in both types of sources, as shown in Table 3. Among news articles, the intentional creation of the virus or its lack of danger were the most repeated narratives, together with useless or dangerous remedies being effective to fight the virus. Interestingly, a more detailed analysis of the cases coded as “Other” narratives showed that a relevant and recurring narrative was the alleged connection between 5G mobile technology and COVID-19.

**Table 3.** Presence of misinformation narratives in the sample.

Recurrent narratives	News articles (n=698), n (%)	Twitter posts (n=1333), n (%)	
The virus has been intentionally created	116 (16.6)	0 (0)	
The virus does not exist or is less harmful that pretended	115 (16.5)	40 (3)	
Useless or dangerous remedies are effective	108 (15.5)	9 (0.7)	
Bill Gates is involved in some way with the virus, the measures, or the vaccines	62 (8.9)	0 (0)	
Vaccines are intentionally harmful or evil	58 (8.3)	5 (0.4)	
The pandemic is part of larger scheme or conspiracy	47 (6.7)	0 (0)	
Vaccines do not work or are not sufficiently tested	37 (5.3)	208 (15.6)	
Lockdowns or other measures are used to control the population or to limit their freedom	29 (4.2)	12 (0.9)	
George Soros is involved in some way with the virus, the measures, or the vaccines	11 (1.6)	0 (0)	
Other narratives			
All other narratives	15 (2.1)	133 (10)	
Related to the connection between 5G and the pandemic	15 (2.1)	0 (0)	
Related to the vaccination process (locations, the relationship of vaccination to other measures, or the need for booster vaccines)	0 (0)	42 (3.1)	
Related to testing (compulsoriness, locations, relationship to other measures)	0 (0)	40 (3)	
Related to the implementation of measures (nonpharmaceutical interventions being in place or not at a given time)	0 (0)	35 (2.6)	
Related to the activity of the public health authorities themselves	0 (0)	10 (0.8)	
Related to warnings about phishing and other cybercrimes	0 (0)	6 (0.5)	
No recurring narrative specified	330 (47.3)	966 (72.5)	

In the case of the Twitter posts, recurring narratives were clearly focused on vaccines. The category of “Other” narratives was quite frequently present, usually due to more specific narratives within the predesigned recurring stories. Some of those narratives related to testing (mostly, the lack of effectiveness of the tests) or to the vaccination process (eg, claims about not needing booster doses of the vaccine; this vaccination process category could be considered a particular narrative connected to those of vaccines not working or being harmful). Other recurring themes identified during the study related to the activity of the studied public health authorities (eg, the workers appearing unexpectedly in houses) or to warnings about scams or phishing in the context of the pandemic. Given the relevance and abundance of these categories, they were added to the predesigned codes for a more detailed observation, as shown in Table 3.

In 47.3% (330/698) of the news articles and 72.5% (966/1333) of the Twitter posts, no recurring narrative was explicitly mentioned. This is significantly higher than the one observed in relation to the topics. This is reasonable, given the higher specificity of narratives and because misinformation could circulate around a diversity of topics that were not always connected to recurring narratives.

Given the connection between topics and narratives, it is no coincidence that vaccine-related narratives were predominant while vaccines were also dominant as a topic. To test that relation between topics and narratives, complementary analyses were conducted, observing the correlation between the presence of topics and narratives. The significant correlations (*P*<.001) are available in Multimedia Appendix 3.

For the news articles, the biggest effect sizes were found in pairs of variables that would be expected to be related: The topic on the origin of the virus had a strong positive correlation with the narrative on the intentional creation of the virus *(r*_696_=0.849, *P*<.001); the topic on the danger and spread of the virus was similarly correlated with the narrative of the virus not existing or being less harmful than pretended by the official discourse *(r*_696_=0.826, *P*<.001); and the topic on pharmaceutical measures other than vaccines had a strong positive correlation with the narrative on the effectiveness of useless or dangerous remedies (*r*_696_=0.839, *P*<.001). It was also interesting to observe some cross-cutting narratives, like the involvement of Bill Gates on the pandemic or the pandemic being part of a larger scheme, that were positively correlated with a diversity of other topics and recurring narratives.

The strongest correlations within the Twitter posts were between the presence of misinformation on technological issues related to the pandemic and warning about phishing and other cybercrimes (*r*_1331_=0.486, *P*<.001) and between the presence of misinformation on social issues related to the pandemic and the recurring narratives related to the activity of the public health authorities themselves (*r*_1331_=0.434, *P*<.001). Contrary to what was observed in the news articles, in which all significant correlations were positive, among the Twitters posts, there were several negative correlations. The strongest was between the presence of misinformation on vaccines and the presence of misinformation on pharmaceutical measures other than vaccines (*r*_1331_=−0.418, *P*<.001).

### Temporary Evolution of Topics and Narratives Present in Pandemic-Related Misinformation

**Figure 2.**
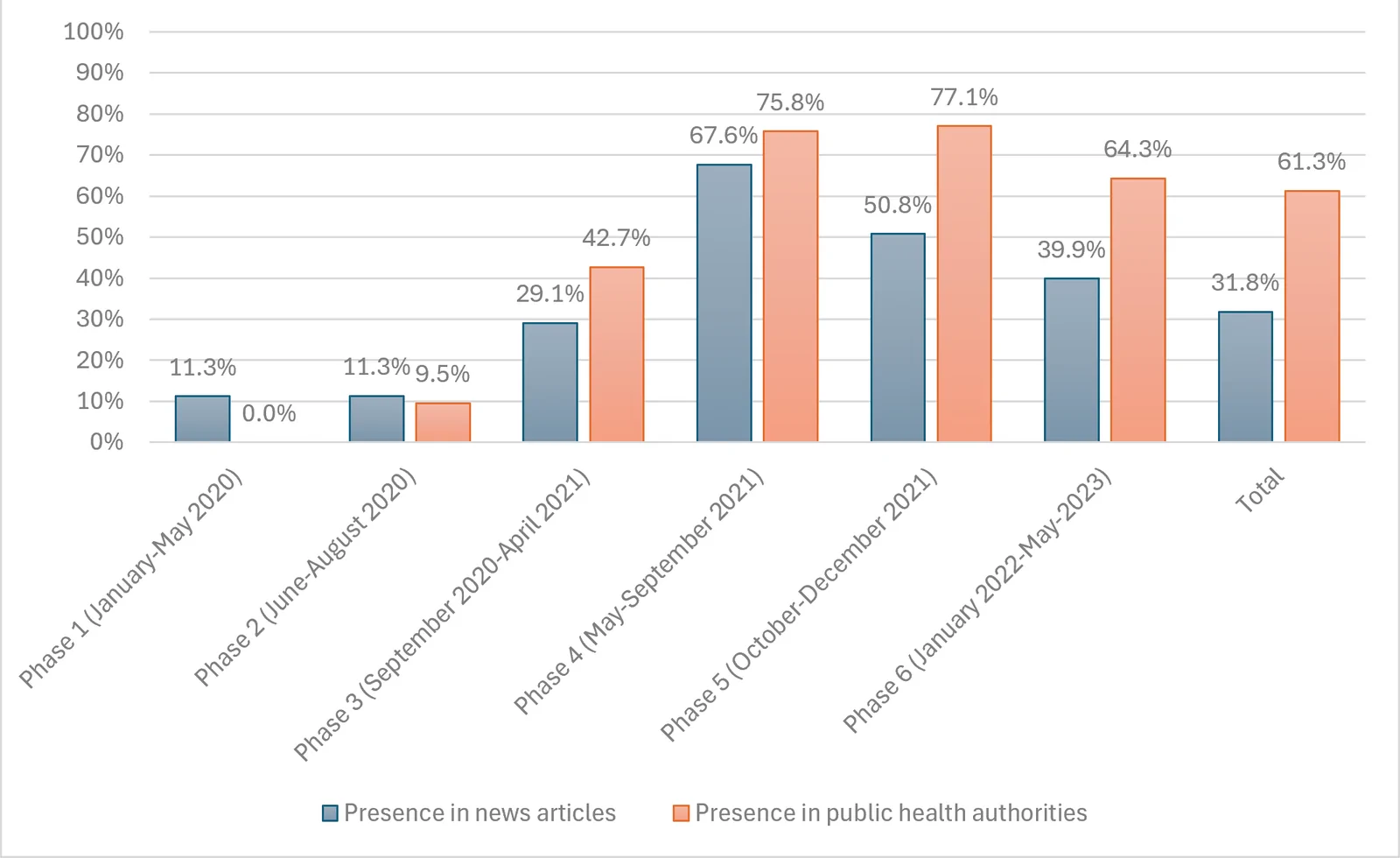
Percentage of studied cases addressing misinformation on vaccines in the 2 studied types of sources across the different phases of the pandemic.

**Figure 3.**
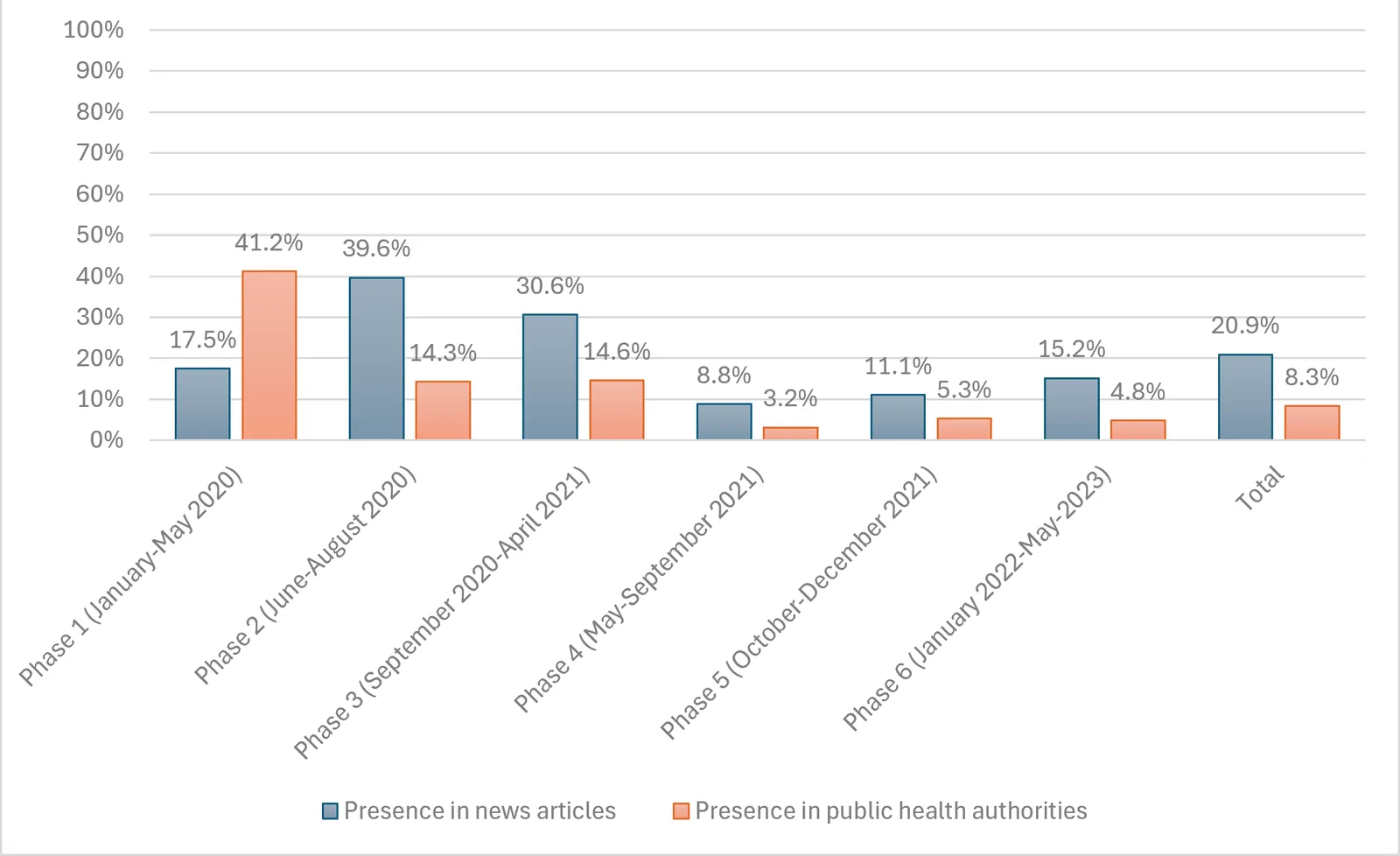
Percentage of studied cases addressing misinformation on the danger and spread of the virus in the 2 studied types of sources across the different phases of the pandemic.

**Figure 4.**
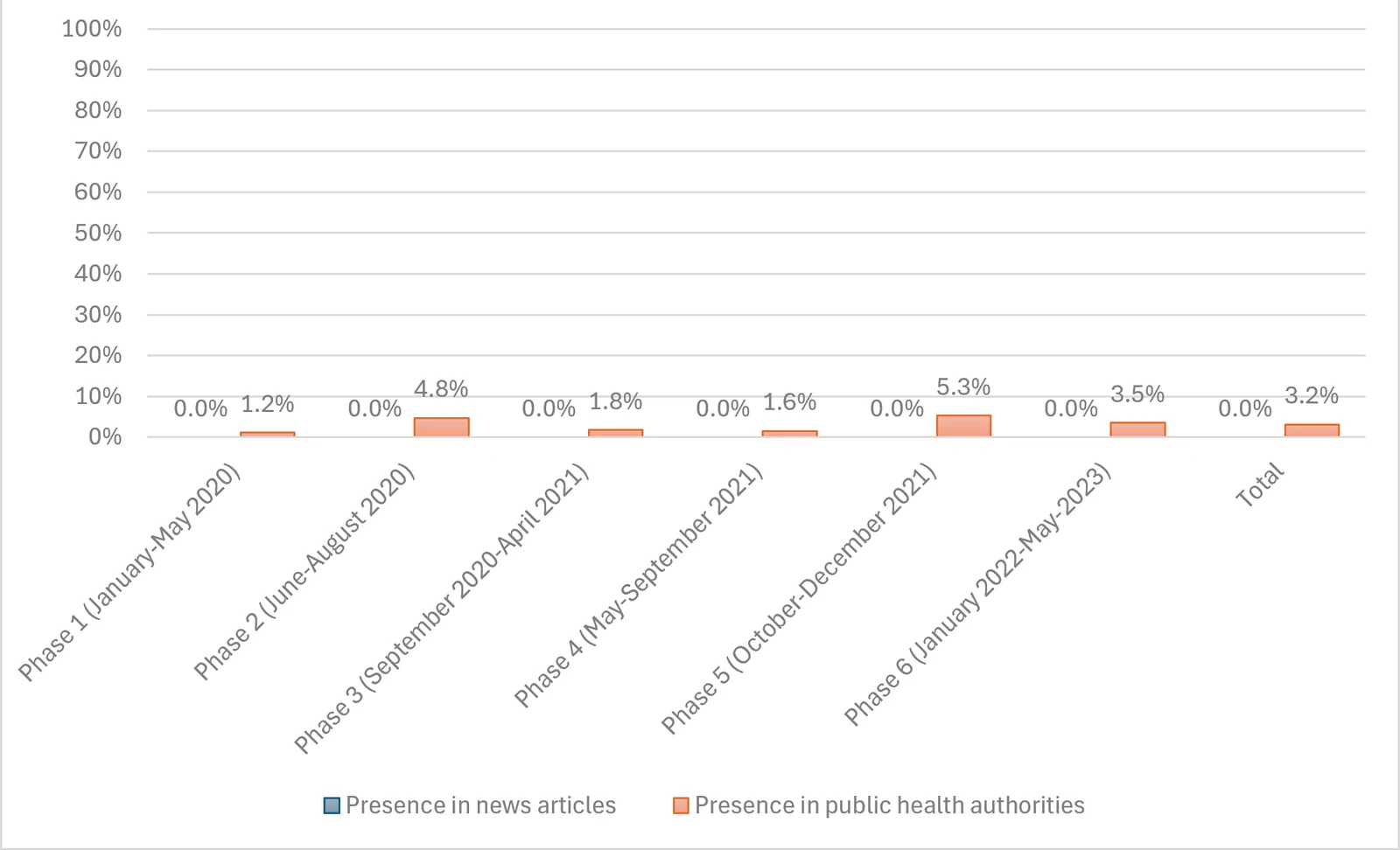
Percentage of studied cases addressing misinformation on the recurring narrative related to the vaccination process in the 2 studied types of sources across the different phases of the pandemic.

The third RQ asked about the potential differences in the presence that the studied topics and narratives had during the different pandemic phases. To study this, *χ*² tests were performed, comparing the mean presence of each topic and narrative over the 6 phases. In general terms, patterns in both types of sources were similar, and most variables showed statistically significant differences across phases. This means that the misinformation content that news media and Twitter accounts of public health authorities decided to deal with varied significantly over the pandemic. Some relevant patterns were the higher presence of misinformation related to the danger and spread of the virus, to its origin, and to the different measures to counter it (including the specific narratives related to these topics) at proportionally higher rates in the first phases, when there was less certainty and more rumors about these issues. In contrast, misinformation on the vaccines increased with time. As an example, Figures 2–4 show the distribution across the topics and narratives with the highest effect size (ie, Cramer V) and therefore a strongest change across different phases: the topic of misinformation on vaccines, the topic of misinformation on the danger and spread of the virus, and the recurring narrative related to the vaccination process that emerged during the analysis (only for the Twitter posts). The figures showing the evolution of the presence of the rest of variables as well as the detailed results of the *χ*² tests are available in Multimedia Appendix 4.

The 2 misinformation topics presented in Figures 2 and 3 had strong changes across the pandemic, albeit in different directions. Vaccine misinformation gained presence as the pandemic advanced, peaking in phases 4 and 5 during the second half of 2021 when the vaccination campaign was in full swing in the Netherlands. This is also the case for the recurring narratives on the vaccines not working or being insufficiently tested and the vaccines being intentionally harmful (Figure 2). In contrast, misinformation on the danger and spread of the virus was more present in the beginning of the pandemic, and it became less relevant in the last phases (Figure 3).

Figure 4, which focuses on misinformation narratives related to the vaccination process (locations, the relationship of vaccination to other measures, or the need for booster vaccines), shows strong fluctuations across phases but without a clear trend; this could be explained by the comparably lower number of cases, which allows for a few instances to have larger impacts on the comparisons, as well as by the spread of specific rumors, such as the discovery of a vaccine when it was not the case (during Phase 2) or the lack of importance of booster vaccines (common in phase 5). Notably, this narrative was only present among public health authorities because it generally focused on the practical aspects of the process, something that specifically concerned the public health authorities involved in the vaccination campaign.

## Discussion

Our study explored the content of misinformation addressed by news media and public health authorities in the Netherlands. By identifying the presence of topics and recurring narratives, it was easier to obtain a clearer picture of the type of false information that the 2 types of studied sources considered more relevant.

The first research question explored the topics of the misinformation cases with which the studied sources dealt. The main topic was the vaccines in both types of sources. Other than vaccines, news media also paid attention to misinformation on the origin, spread, and dangerousness of the virus as well as pharmaceutical measures other than vaccines. In the case of public health authorities, there was less diversity in the addressed misinformation topics, with a particularly strong focus on vaccines and on other measures to control the pandemic, either pharmaceutical, such as medicine and remedies, or nonpharmaceutical, such as lockdowns and tests.

The second research question sought to go beyond topics, digging into more specific recurring narratives. In the news articles, the intentional creation of the virus and the claims about the virus not existing or not being as dangerous were the most abundant. Misinformation narratives regarding specific remedies against the virus (eg, the use of hydroxychloroquine or some natural remedies without a scientific basis) or about the evil behind the vaccines were also frequent. In the case of the public health authorities, the most common recurring narrative focused on the potential harms of the vaccines but without focusing on the intentional evil or harm behind them.

Even if it was not the goal of our study to comprehend the mechanism through which the studied actors decided to address a pandemic-related misinformation case or not, the findings needed to be interpreted in light of the broader, bidirectional dynamics between institutional actors and the public. Prominent misinformation narratives shared on social media among citizens can shape which topics journalists choose to cover and which claims public health authorities decide to address. Thus, the focus on vaccine-related topics and narratives by both sources aligns with existing research that found vaccines were a main misinformation topic [9,11]. Because there is evidence that false information can have agenda-setting capacities [28], it is reasonable that the topics and narratives in Dutch news media and public health communication do not emerge in isolation but are partly responsive to misinformation circulating among the public. However, this dialogue with existing misinformation is not only explained by the abundance of a case of misinformation, as in the case of vaccine misinformation [46], but also by its implications for public health [29]. For instance, a May 2021 Eurobarometer found that misinformation could make people delay or even reject vaccination against COVID-19 [47], and other studies have confirmed the role of misinformation in reduced vaccination uptake [6]. Thus, the potential harm of misinformation to society and to the pandemic response, together with the prominence of a misinformation case in the public and social media discourse, is likely to be a key driver of the choice of institutional actors to address said misinformation case. Future studies are encouraged to further interpret these choices.

More specific observations can be made for each of the studied sources. Regarding news media, their attention to misinformation on the origin and spread of the virus, its remedies, and measures to counter it was also identified in a previous report on misinformation in 2020 [48]. Both the topics and narratives tended to be consistent with some of the most frequent conspiracy theories identified in previous studies. This includes storylines like the alleged involvement of Bill Gates or the connection between the virus and 5G mobile technology [49,50]. On the other hand, public health authorities paid more attention to stories that affected the process of vaccination, testing, and application of measures. One of the reasons behind this is that the studied public health organizations were in charge of the implementation of these measures. As shown in a previous interview study with information intermediaries that included local public health authorities [44], the choices regarding what cases of misinformation to deal with was influenced by the activity and goals of the organization.

It should be noted that, although we differentiated between topics and narratives, there were strong correlations between some of them. For instance, for cases in which a vaccine-related narrative (eg, vaccines are intentionally harmful or evil or vaccines do not work or are not sufficiently tested) was present, the vaccine topic was present as well. The same applied to the narratives on the intentional creation of the virus, which were strongly correlated with the topic on the origin of the virus. Although some topics and narratives were connected by definition, this does not mean that narratives exclusively aligned within one topic; for instance, some of the narratives connecting the pandemic with larger conspiracy schemes or accusing public persons like Bill Gates of their role in the pandemic touched upon the topics of the origin of the virus, the vaccines, and political aspects. In total, our analyses also showed which narratives were most often used in relationship to some topics; this combined observation of topics and recurring narratives offers a more complete picture of the misinformation that the studied sources addressed during the COVID-19 pandemic.

To obtain a comprehensive picture, the third research question of the study sought to go beyond the identification of misinformation topics and narratives and explored the time differences in the distribution of misinformation cases present in news outlets and Twitter posts of public health authorities. In general terms, news media showed a peak of attention to pandemic-related misinformation during the first months of the pandemic when the stories about the origin of the virus, its spread, and the ways to combat the disease were particularly relevant. On the other hand, public health authorities were most active during the year 2021 and beginning of 2022, a period in which the vaccination process was in full swing. More specifically, this is the time in which the vaccine boosters were distributed, which received specific attention as certain narratives argued that they were not needed.

Both the news outlets and the public health authorities paid attention to different topics over different periods of time. The clearest examples are the the higher rates of topics and narratives related to the origin and danger of the virus as well as the measures and strategies to counter it in the early stages, while vaccine-related misinformation was proportionally more present after 2021 in the third and fourth phases of the pandemic. These observations generally concur with those of past research [11], although we managed to combine and correlate topics and narratives, going beyond previous, more descriptive studies. There is, however, room for a deeper understanding of the temporary evolution of the pandemic-related misinformation cases addressed by the studied actors. Our study identified that attention to different topics and narratives among news articles and Twitter posts of public health authorities varied during the COVID-19 pandemic; future research could link this time-varying attention to indicators of public information-seeking and online engagement (eg, search queries, social media activity) for the same narratives, following the approach by Southwell et al [51] of relating virus outbreak news coverage to patterns in online behavior.

To adequately interpret the findings of the study, it is important to consider the different approach to misinformation by news media articles and the Twitter accounts of public health authorities. As summarized in previous paragraphs, some points of convergence emerged, the clearest being the notable attention devoted to vaccine-related misinformation and the similar trends across time. These patterns indicate a connection between the 2 types of sources, not only because they both based their activity on existing circulating misinformation but also because risk communication strategies used by health agencies can influence news media coverage [52] and because public health authorities are common sources of information for journalists covering health topics, especially during pandemics [53,54].

Despite these similarities, there were important divergences in how each type of source addressed misinformation based on their distinct social roles. Journalists tended to examine the impact of misinformation on an issue, covering a wider range of themes with greater depth. This enabled news media to have more diversity in terms of misinformation topics and narratives. Such coverage aligns with the findings of Blanco-Herrero et al [55], who interviewed journalists and identified the level of widespread and newsworthiness of a misinformation case as the main motivators for journalists to deal with them. Thus, they would focus more often on larger news stories, usually with a political and international dimension (eg, the vaccine is evil or the virus is created to control us), as they seek to inform about newsworthy events and developments in society.

In contrast, public health authorities focused on more practical dimensions and decisions that could be affected by misinformation (eg, this measure is now in place, how and when the second dose of the vaccine was needed or distributed), as their communication strategy is connected to their function, which is the promotion of public health and the implementation of measures to counter the effects of the health crisis. Unlike news media, who could cover a diversity of topics based on what was considered newsworthy or relevant for the audience, these authorities limited the use of their Twitter accounts to face rumors, doubts, or questions that could be motivated by misinformation and that affected their usual activity and objectives, without tackling elements that went beyond these areas. Misinformation was rarely explicitly mentioned. Rather than confronting or dissecting it, the studied authorities tended to provide additional information to counter the effects of rumors and misbeliefs.

Two additional factors also shaped these differences. First, platform characteristics determined the style, format, and size of the messages: News articles were longer textual pieces produced by professional journalists; meanwhile Twitter posts were shorter, could include multimedia elements, and were produced by the communication teams of the institutions. Second, our sampling criteria differed: News articles were selected when pandemic-related misinformation was a central element of the text, irrespective of how this misinformation was addressed, which differs from the strategy used in sampling the Twitter posts, where a case was considered when it could be used to counter misinformation even if that was not the sole purpose and there was no direct mention of misinformation. As explained in the methodology, this broader inclusion criterion was applied to public health authorities’ Twitter posts to capture a representative and analytically viable sample of their communication. Applying the more restrictive criteria used for news media would have drastically reduced the dataset, limiting the ability to observe patterns and draw meaningful conclusions.

Although these different sampling strategies were justified by the need to obtain a relevant perspective of the studied materials, they did not allow for valid comparisons between the 2 types of studied sources. Thus, we did not conduct these comparisons in our study. The differences between both types of sources were addressed only for a more complete interpretation of the results.

Another limitation is the study’s inability to determine the precise reasons why a topic or narrative was mentioned. More in-depth qualitative interview studies that use this content analysis as a basis are needed to understand these decision-making processes. Finally, the study’s observations are valid for the Dutch setting, and the findings might not hold true for other national settings. Although replication studies in other countries are recommended, our research did not significantly differ from previous investigations with more limited time frames or different sources [9,11] or less focused on misinformation [40]. Arguably, the Netherlands does not constitute an outlier in the way the COVID-19 pandemic was addressed [36].

Nonetheless, these limitations do not undermine the core aim of the research. By incorporating 2 distinct types of sources, this study offers a broader perspective on the topics and narratives present in misinformation than previous studies focusing on a single source type, often fact checks [20,30]. Our approach yielded a more comprehensive picture of the misinformation addressed by key information sources in the Netherlands during the pandemic, demonstrating the predominance of vaccine misinformation, the fluctuating nature of misinformation during a crisis, and the different approaches to it that different information sources adopt.

## Supplementary material

10.2196/91743 Multimedia Appendix 1 Codebook for news articles.

10.2196/91743 Multimedia Appendix 2 Codebook for Twitter posts.

10.2196/91743 Multimedia Appendix 3 Correlations between the presence of different topics and narratives.

10.2196/91743 Multimedia Appendix 4 Chi-square tests and figures.

## Abbreviations

GGD: Gemeentelijke Gezondheidsdienst
ICR: intercoder reliability
PABAK: prevalence-adjusted and bias-adjusted kappa
RIVM: National Institute for Public Health and the Environment
RQ: research question
WHO: World Health Organization
