# Supplementary material for: Misinformation Through the Lens of Dutch News Media and Public Health Authorities: Content Analysis of Topics and Narratives Present in Pandemic-Related Misinformation During COVID-19
Source: JMIR Infodemiology. 2026 Sep 4;6:e91743. doi: 10.2196/91743 (PMC13544416; doi:10.2196/91743)
Supplement: Supplementary file 1 [file infodemiology-v6-e91743-s001.pdf]

# Codebook for complete content analysis[1]

## Identification

Before starting the coding, read the complete article once. Already during this first reading it might help to highlight key elements that will be later asked in the coding form, such as sources, actors, or topics. The key terms used to select the articles that relate to pandemic-related misinformation are underscored and may be a hint about where the key information is present, but the whole article needs to be read. Keep in mind that the last lines of a text from NexisUni are sometimes captions or connectors (e.g., "see more on page XX"). In the case of online news, the last part of the document includes comments or other elements of the website that are not relevant for the analysis.

1. Identification of the news piece. Alphanumeric variable. Introduce the code of the article. For printed media: file name_number; for online media, file name.
2. Coder. List of options with the identification code for each coder

## General features of the news story

3. Type of medium. Printed media are in the Nexis files, and online media are in independent files.
   a. Printed
   b. Online
4. Medium. Select the medium where the article was published. [Based on what is selected in Q3., only printed/online media will be displayed]
   a. NU.nl
   b. NOS.nl
   c. Algemeen Dagblad
   d. Het Financieele Dagblad
   e. Nederlands Dagblad
   f. NRC
   g. Reformatorisch Dagblad
   h. De Telegraaf
   i. Trouw
   j. de Volkskrant
5. Date of publication. Numeric variable with format DD/MM/YY. In the case of online media, if the article has been updated, only the first date of publication is considered.
6. Number of signing authors. Numeric variable. It is indicated at the top of the article. If the article has no signing author (e.g., editorials) or in the case of news briefs replicated from news agencies, write "0". Note that we only consider authors of the news article; authorship of images (sometimes also appear in the byline) are not considered here.
7. Signing author. Alphanumeric values, authors separated by ;. [Show only if the answer in Q6 is > 0.]
8. Length. Number of words. Numeric variable. In the case of newspapers, it is indicated by Nexis. In the case of online articles, you can copy the text of the article, including the headline and subheadings, in a Word document to count the words.
9. Centrality of **pandemic-related misinformation**. Select only one option. It is important to keep in mind that we refer to pandemic-related misinformation, so mentions to the pandemic alone or to misinformation alone are not considered relevant for our study.

[1] Highlighted in yellow, the variables used for this study

a. Central presence. Misinformation related to the pandemic is the main topic, or it has equal presence with another topic as the most relevant. It is probably mentioned in the heading or the first paragraph.
b. Secondary presence. Misinformation related to the pandemic is connected to the main topic, but it is not dominant. It can, but is not necessarily mentioned in the heading or first paragraph, although it should be mentioned more than once along the text.
c. Contextual or marginal presence. Misinformation related to the pandemic is mentioned only as a contextualization of a character or topic, but not as an issue on its own. Presence is only marginal, with one or few mentions.

## Features of pandemic-related misinformation

All articles are expected to address pandemic-related misinformation, sometimes as an abstract concept (e.g., claiming that misinformation is a risk), others by mentioning more specific cases of pandemic-related misinformation (e.g., explaining that somebody has made a false claim about the coronavirus and how that affects other people). Variables 10-13 focus only on pandemic-related misinformation; mentions to geographic dimensions, topics, or actors that do not refer to pandemic-related misinformation are not considered here.

10. Geographic dimension of the **pandemic-related misinformation** (if a geographical dimension is mentioned, but not in connection to pandemic-related misinformation, it would not be considered here). Select only one option.
    a. Domestic. About the Netherlands, without mentions to any other country.
    b. Domestic, with foreign dimension. Mostly about the Netherlands, but specific reference is made, at least, to some other country, e.g., through actors or sources.
    c. International, with domestic dimension. Mostly about other countries, but specific reference is made to the Netherlands, e.g., through actors or sources.
    d. Foreign. About one or more countries, but without any reference to the Netherlands
    e. It is not clear/Cannot tell. No geographical dimension is mentioned, and there are no mentions to actors or topics that could hint it.
11. Topic of the **pandemic-related misinformation** (if a topic is mentioned, but not in connection to pandemic-related misinformation, it would not be considered here). Select at least one option. More than one option can be selected, as long as the pandemic-related misinformation on this topic is substantially present (it is mentioned or indirectly referred more than once). It does not count if it is only mentioned as a point of comparison or to give context.
    a. Misinformation on the origin of the virus (e.g., whether it was intentional or non-intentional, created in a lab, bat soup in a market in Wuhan, etc.)
    b. Misinformation on the danger and spread of the virus (e.g., exaggerations leading to fear, but also underplaying to make it look like nothing, or even denying the existence of the virus, etc.)
    c. Misinformation on vaccines (e.g., the science behind them, the vaccination campaigns, chips, lack of efficacy, side-effects, etc.)
    d. Misinformation on health-related measures, other than vaccines (e.g., lack of efficacy of antigen tests and facemasks, remedies that worked and didn't, side-effects, dangers of measures, hygiene, etc.)
    e. Misinformation on non-health-related measures (e.g., measures like lockdowns, states of emergency, etc.)

    f. Misinformation on political issues related to the pandemic (e.g., parliamentary discussions, declarations of politicians, elections during the period, etc.)
    g. Misinformation on economic issues related to the pandemic (e.g., unemployment, bankruptcies, economic recovery after the lockdowns, etc.)
    h. Misinformation on social issues related to the pandemic (e.g., effects on coexistence, criminality, immigrations, cases affecting famous people, etc.)
    i. Misinformation on technological issues related to the pandemic (e.g., connections to 5G, tracking apps, etc.)
    j. Misinformation on other pandemic-related topic. Please, specify:
    k. Not specified. Misinformation is discussed as an abstract element and no topic is mentioned (this option is exclusive and cannot be selected together with others).
12. Recurring themes or narratives of the **pandemic-related misinformation**. This variable refers to more specific pandemic-related narratives that have been frequently connected to misinformation. Q10 refers to broad topics, this refers to specific narratives, sometimes across topics, that have been common during the pandemic. Select, when applicable, one or more options, as long as the theme is substantially present (it is mentioned or indirectly referred more than once). It does not count if it is only mentioned as a point of comparison or to give context.
    a. The virus has been intentionally created (no matter by whom)
    b. The virus does not exist or is less harmful than pretended
    c. Lockdowns or other measures are used to control the population or to limit their freedom
    d. Useless or dangerous remedies (e.g., hydroxychloroquine)
    e. Vaccines do not work or are not sufficiently tested
    f. Vaccines are intentionally harmful or evil
    g. The pandemic is part of larger scheme or conspiracy theory (e.g., great reset, great replacement, etc.)
    h. Bill Gates is involved in some way with the virus, the measures or the vaccines
    i. George Soros is involved in some way with the virus, the measures or the vaccines
    j. Other recurring themes or narratives that you have identified during the coding but are not present among the previous options. Please, specify:
    k. None of the above, or not specified (this option is exclusive and cannot be selected together with others).
13. Actors involved in the creation or spread of **pandemic-related misinformation**. This refers to the actors (individuals, groups, or organizations) creating or spreading misinformation: they do not need to be the main spreaders or creators, but it has to be clear in the news piece that they contributed to the spread of pandemic-related misinformation in any way (if an actor is mentioned, but not in connection to pandemic-related misinformation, it would not be considered here). We are not thinking here about actors in the article who do not create or spread misinformation, so exclude, for instance, mentions to victims, subjects of the misinformation case, or sources of real information. Select, when applicable, one or more options.
    a. Public, State, or governmental actors. It includes public figures, institutions, and official communications (RIVM, or EU, for instance). The Prime Minister or other Ministers are included here when they act as public governmental officials (for instance, in a press conference announcing new measures), but they should be coded as political actors when they act as political figures (for instance, in a rally of their party). Note that, even if they are public organizations, health, educational and legal

actors belong to different coding categories. International organizations with a political focus (UN, EU, etc.) would be also included here.

b. Political actors. It includes individual politicians, as well as political parties. The Prime Minister or other Ministers are included here when they act as political figures (for instance, in a rally of their party), but they are considered Public or governmental actors if they act as public officials (for instance, in a press conference announcing new measures).
c. Supranational institutional actors. It refers to international organizations without a political focus. The World Health Organization, UNESCO, UNWTO, etc. belong in this category.
d. Journalistic and media actors. Including individual journalists, and media of any type (e.g, "the Volkskrant wrote that…..".
e. Health or medical actors. Including healthcare professionals and organizations, both public and private.
f. Educational or academic actors. Including professors, researchers, as well as academic or scientific institutions.
g. Legal actors. It includes official legal institutions (e.g., the Supreme Court) and legal companies, including their representatives and professionals such as lawyers, judges, notaries, and other court-related individuals.
h. For-profit organizations or companies. Of any sector or field, including multinationals. It also includes spokespeople and representatives of these organizations.
i. Civil society actors. Lobbies, non-for-profit organizations, NGOs, trade unions, religious organizations, and other similar groups, including those created specifically in connection to the pandemic, as well as those with broader agendas. It also includes spokespeople and representatives of these groups.
j. Public figures. Popular characters not belonging to the previous groups, like celebrities, and actors from the entertainment, sports, or culture fields. They need to be known by the general Dutch public for a reason other than their social media activity (thus, influencers would belong in the next category, no matter how famous they are).
k. Individual citizens or social media actors. They can be regular citizens identified by name (for instance, in an interview or a demonstration) or social media actors and influencers, identified by name or by a pseudonym or username (for instance, an Instagram handle). This includes activists, for instance, but not organized institutions or groups.
l. Other actors. Please, specify:
m. None of the above, or not specified (this option is exclusive and cannot be selected together with others).

## Strategies used in relation to pandemic-related misinformation

All articles are expected to address pandemic-related misinformation. The following questions focus on how they do that: first, regarding the mentions about uncertainty in general, and then focusing on the strategies and sources used in relation to pandemic-related misinformation.

14. Is there any mention about uncertainty or about how the information reported in the article and the facts might be uncertain or not confirmed? The journalist or some source need to state that there is not enough certainty, consensus, or evidence about some of the claims they are making. For instance, it can be about a scientific element (whether a vaccine is safe,

or a facemask works), about what the Government will do, or about whether the claims of a group are certain. Using expressions like "it is said" or "it could be" is not enough; an explicit mention about how something is not known for certain is needed. With just one mention, no matter where in the text, it would be enough. Select only one option:

   a. Yes
   b. No

15. Strategies to report on or deal with **pandemic-related misinformation.** For each of the strategies, select whether it is "not present" or "present" at least once. Keep in mind that more than one strategy can be present.
    a. Acknowledgement. It is stated that that a case (or cases) of pandemic-related misinformation exists, and the false information is stated (e.g., some people are claiming this). Without an explicit mention of the false claim, it is not considered present. It also needs to be made clear that the statement or information is false. Although not essential, it is possible to select this together with other options. Select "present" or "not present".
    b. Analysis. A case (or cases) of pandemic-related misinformation is explained and interpreted (e.g., features, who is behind and why, potential effects, targets…). No matter how small the explanation or the mention of any feature of the misinformation case; it is enough if one of these elements is present for this category to be present. This is, the analysis can go from a broad interpretation over several paragraphs to just one sentence. The false information might be stated or not, so it could appear together with the previous option or not. It can also appear together with other options. Select "present" or "not present".
    c. Correction. A case (or cases) of pandemic-related misinformation is corrected, claiming that false information is circulating about a topic, and what the truth is (e.g., this case of misinformation is false because of this statistic, which shows that the truth is this other one). This could be also done without stating the false claim (e.g., there is misinformation about this topic, but the truth is this one). It can also appear together with other options. The key aspect is the presence of the correct information in opposition to false claims. Mentioning just correct information without referring to the existence of false claims would not be considered here. Select "present" or "not present". [Q16, Q17 and Q18 are shown only if C is marked in Q15.]
    d. Prebunking. It seeks to prevent the effects of pandemic-related misinformation. The goal is to make people aware of the problem or of potential techniques and narratives used in misinformation, usually with an educational approach (e.g., warning that some types of social media messages are not reliable and might include misinformation). A raising awareness or educational aspect has to be explicit. It can appear together with other options. Although it can refer to a specific misinformation case, it has to have a broader dimension and offer tips or explanations valid for more cases. Select "present" or "not present".
    e. Discussion. Pandemic-related misinformation is discussed as a broader topic, going beyond a case or cases (e.g., explaining how pandemic-related misinformation has influenced some elections, alarming about its risks, arguing that misinformation is a big or a small problem). If this discussion is connected to a specific case, it would be option b. Option e is selected only when it tackles broader aspects. This does not mean that it cannot appear together with other options in the same article, but it has to be clearly differentiated and have no relationship with any specific misinformation case. Select "present" or "not present".

    f. Other strategies. Please, specify:

16. Depth of the strategies to correct the pandemic-related misinformation case or cases. When only one correction is present, only one option can be selected; if more than one case of pandemic-related misinformation is corrected and different strategies are used, you can mark both options.
    a. Simple rebuttal. Brief correction, using fewer arguments in refuting the false information. The correction includes true statements without further elaboration and is done in maximum one paragraph (probably even less) using brief sentences (e.g., this is false, the truth is this). Using one source (this is true because this source claims it) is considered simple, but using more sources or adding extra information would be already factual elaboration.
    b. Factual elaboration. A detailed debunking message with more elaborated narratives and detailed descriptions about the facts. The correction includes true statements that are further explained, usually over more than one paragraphs (e.g., this is false, the truth is this because… Furthermore...).
17. Strength of the strategies to correct the pandemic-related misinformation case or cases. Level of uncertainty of the information used to correct the pandemic-related misinformation case or cases. Note that Q14 also referred to uncertainty, but here the reference to uncertainty has to be made as part of the correction, and other mentions in the text are not considered. When only one correction is present, only one option can be selected; if more than one case of pandemic-related misinformation is corrected and different strategies are used, you can mark both options.
    a. Hedged. The correction indicates uncertainty, either because the false statement is difficult to correct, or because the existent true information is uncertain or incomplete (e.g., according to some sources, this could be false…). Both the false and the true information can be stated, but the journalist is not completely sure about the assessment.
    b. Direct. The correction does not indicate uncertainty, and it uses statements without doubting their validity (e.g., based on this/these source(s), this is false).
18. Order of the strategies to correct the pandemic-related misinformation case or cases. When only one correction is present, only one option can be selected; if more than one case of pandemic-related misinformation is corrected and they use different strategies, you can mark both options.
    a. Correction before misinformation. The correct information is shown before the misinformation. It might appear immediately before (e.g., vaccines work, although some fake news say they do not) or in a further place within the article (e.g., the true information is discussed along the text and the false statement is mentioned later to add context).
    b. Correction after misinformation. The correct information is shown after the misinformation. It might appear immediately after (e.g., some fake news say that vaccines do not work, but the truth is…) or in a further place within the article (e.g., the false statement is discussed along the text and the true information appears at a later point).
19. Number of **expert** sources mentioned. Numeric value. It refers to the number of different sources (individuals or groups or organizations) used in the article, denoted as specialists or reliable source in their specific area. Witnesses are not considered experts. In most cases, experts might be presented with the reason why they are experts (e.g., a professor, a doctor, somebody with long experience in a field). We consider an expert source when it is presented

as a reliable credible source due to their expertise or knowledge. For example, if a story is about a politician claiming that misinformation is a risk that his government will fight, they would not be an expert source. Similarly, if a participant in a demonstration is interviewed to understand their view, they do not count as experts. But if a professor is quoted to support what the politician or the citizen said, this professor would be an expert source. Note that we are not evaluating the quality of the source, whether they are independent, or whether they say the truth. We count them as an expert source if they are used as such based on the previous description, that is, they provide information needed to complete the story, but they are not part of it.

20. Type of **expert** sources mentioned. One source cannot be identified as more than one type, but more than one type can be selected when it refers to different sources. It is possible that more than one source belongs to the same type; in that case, it is enough with marking the category once. In total, the different types selected can be equal or lower than the number of sources in the previous question. Note that a source may fall within more than one type (e.g. the Prime Minister could be a State Source, but also a Political Source, depending on their role in the story), but the type should be coded depending on their condition in the specific news story. Exceptionally, if a source is explicitly assigned to two groups (e.g., a member of a civil society group who is also a university professor), more than one category can be selected. Anonymous sources are coded as the type to which they belong. [Present only if the number of experts in Q19 >0.]
    a. Public, State, or Governmental Source. This refers to people or organizations who work administering transnational, national, regional/provincial, or municipal governments. This category contains a wide array of government divisions and employees, including bureaucrats, administrators, representatives, executives, etc. It also includes civil servants working for public institutions, including the police, the military, defense corps or intelligence services. The Prime Minister or other Ministers are included here when they act as public officials (for instance, as responsible for governing decisions), but they are considered Political Sources when they act as political figures (for instance, representing their party). Note that, even if they are public organizations, health, educational and legal sources belong to different categories. International organizations with a political focus (UN, EU, etc.) would be also included here.
    b. Political Source. It refers to political associations or members of these associations. The Prime Minister or other Ministers are included here when they act as political figures (for instance, representing their party), but they are considered State or Government Sources if they act as public officials (for instance, as responsible for governing decisions). Note that, if considering expert sources, this category should be less frequent than the previous one.
    c. Supranational institutional Source. It refers to international organizations without a political focus. The World Health Organization, UNESCO, UNWTO, etc. belong in this category.
    d. Journalistic or media Source. This refers to media outlets (e.g., "the Volkskrant reported…") or to members that speak in representation of them (e.g., the chief editor of Trouw stated that…"), or professional journalists, other than the author of the news story.
    e. Health or medical Source. It refers to different types of health care organizations and their personnel, including, but not limited to, public, or private health care professionals of different specializations.

f. Educational and Academic Source. This category refers to educational organizations at different levels and their personnel, including teachers, faculty members, professors, educational managers, scientists, and researchers from different areas of expertise. The sources are included because of their knowledge of a topic they study.
g. Legal and Court Source. This category includes different types of judiciary personnel, such as judges, magistrates, adjudicators, as well as public legal professionals, prosecutors, state lawyers, public notaries, legal aid officers, and other court-related officials. This category also includes private legal professionals, such as lawyers.
h. Business or for-profit Organization Source. This refers to actors from the commercial sector as well as trade groups. It includes representatives from associations from the corporate sector, companies (public or private), and specific businesspeople.
i. Civil Society Source. It refers to members of an organized civil society, such as an NGO, union, religious groups, or similar social organization, other than the state and business world.
j. Sports, Performance, Artist, or Celebrity Source. This category refers to the broad area of entertainment, including sport people and organizations, as well as celebrities and organizations and individuals working in the areas of music, theater, film, photography, literature, painting, museums, fashion, design, and (non-informative) television shows. They need to be known by the general Dutch public for a reason other than their social media activity (thus, influencers would belong in the next category, no matter how famous they are).
k. Individual Citizens or Social Media Source. This includes people as individual sources of information, and not in representation of anyone, as in the previous categories. This category contains regular citizens, as well as bloggers, instagrammers, YouTubers, and other social media actors. This includes activists, as well as other professionals that do not belong in previous groups (e.g., an architect), but not organized institutions or groups.
l. Other Sources. This includes sources that cannot be assigned to any of the categories listed above. <u>Please, specify</u>:

21. Journalistic roles. For each of the roles, select to what extent it is present. Given that the presence of a role is not a dichotomic choice, you can select for each of the following roles whether they are "not present" (no presence whatsoever), "somehow present" (some aspects of the role might be present, but the role is not predominant in the news story) or "clearly present" (the role is predominant in the news story: you should be able to identify the presence of the role already after the first reading).
    a. Interventionist. **It is present** when there is presence of the journalist's point of view, interpretation, calls for action, or use of qualifying adjectives or first person. The journalist is "present" in the story, either using his/her own voice, explaining the process to collect the information, or offering personal opinions. Although selecting a certain topic or frame can be considered interventionism, the choices that are not explicit in the article are not considered. Thus, using specific sources or information to control, criticize or defend other groups is not necessarily interventionism, the "attacks" or "praises" need to come from the journalist's voice for interventionism to be present.
    b. Watchdog (elites). **It is present** when there is information on judicial or administrative processes against the political, social, cultural or economic elites, when there is criticism to those in powerful positions, or doubts about the validity or truthfulness of their statements, when hidden information about these elites is uncovered, when

external investigations or investigative reporting against them are used, or when conflict between the journalist and those in powerful positions is present. Holding the elite accountable can also imply making hidden information available. Usually, the information or comments of the watchdog role are uncomfortable for some members of the elite; that is, when this role is present somebody would wish that the article would not exist. We consider elites as those with a significant power on a society and capacity to determine the media agenda; thus, in normal conditions, a member of the government is the political elite, but a local politician in a small village is not; the owner of a big multinational company is the economic elite, a small businessperson with a shop in a city is not, the University of Amsterdam as an institution or its rector can be part of the social elite, but a researcher or a doctor are not.

c. Watchdog (citizens). **It is present** when there is information on judicial or administrative processes against regular citizens or civil society groups, when there is criticism to regular citizens or civil society groups, or doubts about the validity or truthfulness of their statements, when hidden information about regular citizens or civil society groups is uncovered, when external investigations or investigative reporting against these groups are used, or when conflict between the journalist and regular citizens or civil society groups is present. This role is a variation of the previous one, but it focuses on ordinary citizens or civil society rather than elites. We consider elites as those with a significant power on a society and capacity to determine the media agenda; thus, in normal conditions, a member of the government is the political elite, but a local politician in a small village is not; the owner of a big multinational company is the economic elite, a small businessperson with a shop in a city is not, the University of Amsterdam as an institution or its rector can be part of the social elite, but a researcher or a doctor are not.
d. Facilitator. **It is present** when there is a defense or support of activities or policies, a positive image of the political, social, or economic elite, when the story portrays progress or success or a positive comparison to other countries, or when there is reporting on national's triumphs, or presence of promotion of country's image or patriotism. Just using official or governmental sources does mean this role is present, there needs to be a clear defense or positive comparison of what has been done by somebody (usually the government or the country as a whole). Reporting on the thing the government or other group of the elite have done is not enough, but becoming channels for the transmission of the elites' messages to the public would be considered facilitator role (unlike the watchdog role, here the government or other members of the elite are happy that the journalist is reporting or transmitting a given information).
e. Service. **It is present** when there are mentions to the impact of measures or events on everyday life, or when the story offers tips and advice about individual risks, consumer information, consumer advice, or personal assistance. The presence of the prebunking strategy in Q15 is a good example of this role. The journalist does not only offer information, but also practical guidance for the daily life of the audience (this includes practical information to face the virus or the lockdown). In general, these are news you can use.
f. Infotainment. **It is present** when there is presence of personalization (the personal feelings and perspective of a character gains relevance within the news event), mentions to private life, sensationalism, or emotions. The journalist does not only want to inform or be useful, but also to entertain and thrill the public. The style and

topics are not associated with hard information, but they are closer to entertainment genres (e.g., action movies, TV dramas, suspense novels) by using story-telling devices and establishing characters and setting.

g. Civic. **It is present** when there are mentions to citizen reactions, citizen demands, credibility of citizens, local impact, or social community impact, when the article educates on duties and rights, or includes contextual background information, citizen questions, information on citizen activities, or support of citizen movements. It differentiates from the service role because here the news are not only (or not necessarily) useful for the daily life, but for the political and civic participation of the citizen. Note that the service role has a more practical approach to the audience, seen as consumers, here the audience is seen as citizens within a community. Although it is not its goal, this role could be considered a complement of the facilitator but instead of acting as transmitter or supporter of the elite, the journalist supports or empowers the citizens. In this role it is more likely to give voice (not necessarily as expert sources, but at least as participants in the story) to actors or institutions without social empowerment who demand recognition or reinstatement of a right.

## Presence of underserved groups

22. Is there any mention about ANY sociodemographic group affected by misinformation. These groups can be based on age, place of living, origin, economic status, educational level, or any other related variable. We care about statements like, for instance, "this story has spread mostly among young people" or "this source is common among migrants with a [country/region] background." It does not matter which kind of group it is, how misinformation might affect them, or whether they are underserved or vulnerable in any sense. We care only if they have been affected by misinformation. With just one mention of any group it would be enough. Select only one option:
    a. Yes
    b. No
23. Mentions of sociodemographic groups affected by misinformation. Alphanumerical variable. Write groups separated by ; and include as many sociodemographic groups as mentioned. [Show only if Yes was selected in Q22]
24. Is there any mention about how some groups have limited access to information or are more vulnerable to misinformation or rumors? Note that in this question it is not enough to have been affected by misinformation, it has to refer to the lack or difficult access of some groups to reliable and mainstream sources of information. The might refer, for example, to people with low literacy levels, living on the street, with migration background who do not speak the language… With just one mention of any group it would be enough. Select only one option:
    a. Yes
    b. No
25. Mentions of groups underserved in terms of access to information. Alphanumeric variable. Write groups separated by ; and include as many groups as mentioned. [Show only if Yes was selected in Q24]
