# Supplementary material for: Misinformation Through the Lens of Dutch News Media and Public Health Authorities: Content Analysis of Topics and Narratives Present in Pandemic-Related Misinformation During COVID-19
Source: JMIR Infodemiology. 2026 Sep 4;6:e91743. doi: 10.2196/91743 (PMC13544416; doi:10.2196/91743)
Supplement: Supplementary file 3 [file infodemiology-v6-e91743-s003.docx]

**Supplementary material – Correlations between the presence of different topics and narratives**

**Table 1**. Significant correlations between the presence of different topics and narratives in the studied news articles

| **Variables** | **Correlation** |
| --- | --- |
| Misinformation on the origin of the virus (Topic) 🡨🡪 Misinformation on technological issues related to the pandemic (Topic) | *r*(696) = .135, *p* < .001 |
| Misinformation on the origin of the virus (Topic) 🡨🡪 The virus has been intentionally created (Narrative) | *r*(696) = .849, *p* < .001 |
| Misinformation on the origin of the virus (Topic) 🡨🡪 The pandemic is part of a larger scheme or conspiracy theory (Narrative) | *r*(696) = .207, *p* < .001 |
| Misinformation on the origin of the virus (Topic) 🡨🡪 Bill Gates is involved in some way with the virus, the measures or the vaccines (Narrative) | *r*(696) = .382, *p* < .001 |
| Misinformation on the origin of the virus (Topic) 🡨🡪 George Soros is involved in some way with the virus, the measures or the vaccines (Narrative) | *r*(696) = .137, *p* < .001 |
| Misinformation on the danger and spread of the virus (Topic) 🡨🡪 The virus does not exist or is less harmful than pretended (Narrative) | *r*(696) = .826, *p* < .001 |
| Misinformation on the danger and spread of the virus (Topic) 🡨🡪 Lockdowns or other measures are used to control the population or to limit their freedom (Narrative) | *r*(696) = .140, *p* < .001 |
| Misinformation on vaccines (Topic) 🡨🡪 Vaccines do not work or are not sufficiently tested (Narrative) | *r*(696) = .333, *p* < .001 |
| Misinformation on vaccines (Topic) 🡨🡪 Vaccines are intentionally harmful or evil (Narrative) | *r*(696) = .396, *p* < .001 |
| Misinformation on vaccines (Topic) 🡨🡪 Bill Gates is involved in some way with the virus, the measures or the vaccines (Narrative) | *r*(696) = .187, *p* < .001 |
| Misinformation on pharmaceutical measures, other than vaccines (Topic) 🡨🡪 Misinformation on non-pharmaceutical measures (Topic) | *r*(696) = .166, *p* < .001 |
| Misinformation on pharmaceutical measures, other than vaccines (Topic) 🡨🡪 Lockdowns or other measures are used to control the population or to limit their freedom (Narrative) | *r*(696) = .139, *p* < .001 |
| Misinformation on pharmaceutical measures, other than vaccines (Topic) 🡨🡪 Useless or dangerous remedies are effective (Narrative) | *r*(696) = .839, *p* < .001 |
| Misinformation on non-pharmaceutical measures (Topic) 🡨🡪 Misinformation on political issues related to the pandemic (Topic) | *r*(696) = .168, *p* < .001 |
| Misinformation on non-pharmaceutical measures (Topic) 🡨🡪 Lockdowns or other measures are used to control the population or to limit their freedom (Narrative) | *r*(696) = .381, *p* < .001 |
| Misinformation on non-pharmaceutical measures (Topic) 🡨🡪 Useless or dangerous remedies are effective (Narrative) | *r*(696) = .150, *p* < .001 |
| Misinformation on political issues related to the pandemic (Topic) 🡨🡪 The pandemic is part of larger scheme or conspiracy theory (Narrative) | *r*(696) = .226, *p* < .001 |
| Misinformation on social issues related to the pandemic (Topic) 🡨🡪 The pandemic is part of larger scheme or conspiracy theory (Narrative) | *r*(696) = .291, *p* < .001 |
| Misinformation on technological issues related to the pandemic (Topic) 🡨🡪 The virus has been intentionally created (Narrative) | *r*(696) = .218, *p* < .001 |
| Misinformation on technological issues related to the pandemic (Topic) 🡨🡪 Bill Gates is involved in some way with the virus, the measures or the vaccines (Narrative) | *r*(696) = .218, *p* < .001 |
| The virus has been intentionally created (Narrative) 🡨🡪 Bill Gates is involved in some way with the virus, the measures or the vaccines (Narrative) | *r*(696) = .361, *p* < .001 |
| Lockdowns or other measures are used to control the population or to limit their freedom (Narrative) 🡨🡪 Vaccines do not work or are not sufficiently tested (Narrative) | *r*(696) = .143, *p* < .001 |
| Vaccines are intentionally harmful or evil (Narrative) 🡨🡪 The pandemic is part of larger scheme or conspiracy theory (Narrative) | *r*(696) = .147, *p* < .001 |
| Vaccines are intentionally harmful or evil (Narrative) 🡨🡪 Bill Gates is involved in some way with the virus, the measures or the vaccines (Narrative) | *r*(696) = .253, *p* < .001 |
| The pandemic is part of larger scheme or conspiracy theory (Narrative) 🡨🡪 Bill Gates is involved in some way with the virus, the measures or the vaccines (Narrative) | *r*(696) = .157, *p* < .001 |
| The pandemic is part of larger scheme or conspiracy theory (Narrative) 🡨🡪 George Soros is involved in some way with the virus, the measures or the vaccines (Narrative) | *r*(696) = .150, *p* < .001 |
| Bill Gates is involved in some way with the virus, the measures or the vaccines (Narrative) 🡨🡪 George Soros is involved in some way with the virus, the measures or the vaccines (Narrative) | *r*(696) = .365, *p* < .001 |

**Table 2**. Significant correlations between the presence of different topics and narratives in the studied social media posts from public health authorities

| **Variables** | **Correlation** |
| --- | --- |
| Misinformation on the danger and spread of the virus (Topic) 🡨🡪 Misinformation on vaccines (Topic) | *r*(1331) = -.240, *p* < .001 |
| Misinformation on the danger and spread of the virus (Topic) 🡨🡪 Other recurring narratives: related to the implementation of measures (Narrative) | *r*(1331) = .205, *p* < .001 |
| Misinformation on vaccines (Topic) 🡨🡪 Misinformation on pharmaceutical measures, other than vaccines (Topic) | *r*(1331) = -.418, *p* < .001 |
| Misinformation on vaccines (Topic) 🡨🡪 Misinformation on non-pharmaceutical measures (Topic) | *r*(1331) = -.263, *p* < .001 |
| Misinformation on vaccines (Topic) 🡨🡪 The virus does not exist or is less harmful than pretended (Narrative) | *r*(1331) = -.158, *p* < .001 |
| Misinformation on vaccines (Topic) 🡨🡪 Vaccines do not work or are not sufficiently tested (Narrative) | *r*(1331) = .342, *p* < .001 |
| Misinformation on vaccines (Topic) 🡨🡪 Other recurring narratives: related to the vaccination process (Narrative) | *r*(1331) = .117, *p* < .001 |
| Misinformation on vaccines (Topic) 🡨🡪 Other recurring narratives: related to testing (Narrative) | *r*(1331) = -.221, *p* < .001 |
| Misinformation on vaccines (Topic) 🡨🡪 Other recurring narratives: related to the implementation of measures (Narrative) | *r*(1331) = -.120, *p* < .001 |
| Misinformation on pharmaceutical measures, other than vaccines (Topic) 🡨🡪 Misinformation on non-pharmaceutical measures (Topic) | *r*(1331) = .131, *p* < .001 |
| Misinformation on pharmaceutical measures, other than vaccines (Topic) 🡨🡪 Useless or dangerous remedies are effective (Narrative) | *r*(1331) = .098, *p* < .001 |
| Misinformation on pharmaceutical measures, other than vaccines (Topic) 🡨🡪 Vaccines do not work or are insufficiently tested (Narrative) | *r*(1331) = -.205, *p* < .001 |
| Misinformation on pharmaceutical measures, other than vaccines (Topic) 🡨🡪 Other recurring narratives: related to testing (Narrative) | *r*(1331) = .279, *p* < .001 |
| Misinformation on pharmaceutical measures, other than vaccines (Topic) 🡨🡪 Other recurring narratives: related to the implementation of measures (Narrative) | *r*(1331) = .269, *p* < .001 |
| Misinformation on non-pharmaceutical measures (Topic) 🡨🡪 Vaccines do not work or are not sufficiently tested (Narrative) | *r*(1331) = -.141, *p* < .001 |
| Misinformation on political issues related to the pandemic (Topic) 🡨🡪 Other recurring narratives: related to the activity of the public health authorities themselves (Narrative) | *r*(1331) = .315, *p* < .001 |
| Misinformation on social issues related to the pandemic (Topic) 🡨🡪 Other recurring narratives: related to the activity of the public health authorities themselves (Narrative) | *r*(1331) = .434, *p* < .001 |
| Misinformation on technological issues related to the pandemic (Topic) 🡨🡪 Other recurring narratives: related to warning about phishing and other cybercrimes (Narrative) | *r*(1331) = .486, *p* < .001 |
| The virus does not exist or is less harmful than pretended (Narrative) 🡨🡪 Useless or dangerous remedies (Narrative) | *r*(1331) = .093, *p* < .001 |
| The virus does not exist or is less harmful than pretended (Narrative) 🡨🡪 Other recurring narratives: related to the implementation of measures (Narrative) | *r*(1331) = .329, *p* < .001 |
| Lockdowns or other measures are used to control the population or to limit their freedom (Narrative) 🡨🡪 Other recurring narratives: related to the activity of the public health authorities themselves (Narrative) | *r*(1331) = .176, *p* < .001 |
| Vaccines do not work or are not sufficiently tested (Narrative) 🡨🡪 Vaccines are intentionally harmful or evil (Narrative) | *r*(1331) = .109, *p* < .001 |
