# Supplementary material for: Misinformation Through the Lens of Dutch News Media and Public Health Authorities: Content Analysis of Topics and Narratives Present in Pandemic-Related Misinformation During COVID-19
Source: JMIR Infodemiology. 2026 Sep 4;6:e91743. doi: 10.2196/91743 (PMC13544416; doi:10.2196/91743)
Supplement: Supplementary file 4 [file infodemiology-v6-e91743-s004.docx]

**Supplementary material – Chi-square tests and figures to respond to RQ3**

**Table 1**. Chi-square tests of the presence of misinformation topics in the studied sample over different pandemic phases

| **Topics** | **News articles** | | **Twitter posts** | |
| --- | --- | --- | --- | --- |
|  | **χ²(5, N = 698)** | **V** | **χ²(5, N = 1,333)** | **V** |
| Misinformation on vaccines (e.g., vaccines include microchips) – Figure 1 | **97.58, p < .001** | **.37** | **235.56, p < .001** | **.42** |
| Misinformation on the danger and spread of the virus (e.g., the virus is not real) – Figure 2 | **36.29, p < .001** | **.23** | **150.64, p < .001** | **.34** |
| Misinformation on the origin of the virus (e.g., the virus was intentionally created) – Figure 3 | **43.64, p < .001** | **.25** | 1.48, p = .92 | .03 |
| Misinformation on pharmaceutical measures, other than vaccines (e.g., hydroxychloroquine kills the virus) – Figure 4 | **29.64, p < .001** | **.21** | **46.43, p < .001** | **.19** |
| Misinformation on technological issues related to the pandemic (e.g., the COVID app is used to track citizens) – Figure 5 | **61.52, p < .001** | **.30** | **18.40, p = .002** | **.12** |
| Misinformation on non-pharmaceutical measures (e.g., the lockdown is used to control the population) – Figure 6 | **16.13, p = .01** | **.15** | **27.09, p < .001** | **.14** |
| Misinformation on social issues related to the pandemic (e.g., immigrants are not respecting the restrictions) – Figure 7 | 7.75, p = .17 | .11 | **92.63, p < .001** | **.26** |
| Misinformation on political issues related to the pandemic (e.g., alleged declarations of politicians) – Figure 8 | 4.52, p = .48 | .08 | 1.48, p = .92 | .03 |
| Misinformation on economic issues related to the pandemic (e.g., the pandemic is destroying a particular economic field) – Figure 9 | 7.24, p = .20 | .10 | - | - |
| Other topics | - | - | - | - |

In bold, statistically significant results at p < .05.

**Figure 1**. Percentage of studied cases addressing misinformation on vaccines in the two studied types of sources across the different phases of the pandemic

**Figure 2**. Percentage of studied cases addressing misinformation on the danger and spread of the virus in the two studied types of sources across the different phases of the pandemic

**Figure 3**. Percentage of studied cases addressing misinformation on the origin of the virus in the two studied types of sources across the different phases of the pandemic

**Figure 4**. Percentage of studied cases addressing misinformation on pharmaceutical measures other than vaccines in the two studied types of sources across the different phases of the pandemic

**Figure 5**. Percentage of studied cases addressing misinformation on technological issues related to the pandemic in the two studied types of sources across the different phases of the pandemic

**Figure 6**. Percentage of studied cases addressing misinformation on non-pharmaceutical measures in the two studied types of sources across the different phases of the pandemic

**Figure 7**. Percentage of studied cases addressing misinformation on social issues related to the pandemic in the two studied types of sources across the different phases of the pandemic

**Figure 8**. Percentage of studied cases addressing misinformation on political issues related to the pandemic in the two studied types of sources across the different phases of the pandemic

**Figure 9**. Percentage of studied cases addressing misinformation on economic issues related to the pandemic in the two studied types of sources across the different phases of the pandemic

**Table 2**. Chi-square tests of the presence of misinformation recurring narratives in the studied sample over different pandemic phases

| **Recurrent narratives** | **News articles** | | **Twitter posts** | |
| --- | --- | --- | --- | --- |
|  | **χ²(5, N = 698)** | **V** | **χ²(5, N = 1,333)** | **V** |
| The virus has been intentionally created – Figure 10 | **46.28, p < .001** | **.26** | - | - |
| The virus does not exist or is less harmful that pretended – Figure 11 | **17.24, p = .004** | **.16** | **58.81, p < .001** | **.21** |
| Useless or dangerous remedies are effective – Figure 12 | **53.41, p < .001** | **.28** | 5.23, p = .40 | .06 |
| Bill Gates is involved in some way with the virus, the measures or the vaccines – Figure 13 | **16.01, p = .01** | **.15** | - | - |
| Vaccines are intentionally harmful or evil – Figure 14 | **16.38, p = .01** | **.15** | 3.95, p = .56 | .05 |
| The pandemic is part of larger scheme or conspiracy – Figure 15 | **14.91, p = .01** | **.15** | - | - |
| Vaccines do not work or are not sufficiently tested – Figure 16 | **13.86, p = .02** | **.14** | **91.24, p < .001** | **.26** |
| Lockdowns or other measures are used to control the population or to limit their freedom – Figure 17 | **12.43, p = .03** | **.13** | **60.81, p < .001** | **.21** |
| George Soros is involved in some way with the virus, the measures or the vaccines – Figure 18 | 3.75, p = .59 | .07 | - | - |
| Other narratives: related to the connection between 5G and the pandemic – Figure 19 | **39.26, p < .001** | **.23** | - | - |
| Other narratives: related to the vaccination process (locations, the relationship of vaccination to other measures, or the need for booster vaccines) – Figure 20 | - | - | **58.51, p < .001** | **.66** |
| Other narratives: related to testing (compulsoriness, locations, relationship to other measures) – Figure 21 | - | - | **31.20, p < .001** | **.48** |
| Other narratives: related to the implementation of measures (which non-pharmaceutical interventions were in place or not at each given time) – Figure 22 | - | - | **26.72, p < .001** | **.45** |
| Other narratives: related to the activity of the public health authorities themselves – Figure 23 | - | - | 8.09, p = .15 | .25 |
| Other narratives: related to warnings about phishing and other cybercrimes – Figure 24 | - | - | **33.51, p < .001** | **.50** |

In bold, statistically significant results at p < .05.

**Figure 10**. Percentage of studied cases addressing misinformation on the recurring narrative related to the virus having been intentionally created (no matter by whom) in the two studied types of sources across the different phases of the pandemic

**Figure 11**. Percentage of studied cases addressing misinformation on the recurring narrative related to the virus not exiting or being less harmful than pretended in the two studied types of sources across the different phases of the pandemic

**Figure 12**. Percentage of studied cases addressing misinformation on the recurring narrative related useless or dangerous remedies in the two studied types of sources across the different phases of the pandemic

**Figure 13**. Percentage of studied cases addressing misinformation on the recurring narrative related to Bill Gates being involved in some way with the virus, the measures or the vaccines in the two studied types of sources across the different phases of the pandemic

**Figure 14**. Percentage of studied cases addressing misinformation on the recurring narrative related to vaccines being intentionally harmful or evil in the two studied types of sources across the different phases of the pandemic

**Figure 15**. Percentage of studied cases addressing misinformation on the recurring narrative related to the pandemic being part of a larger scheme or conspiracy in the two studied types of sources across the different phases of the pandemic

**Figure 16**. Percentage of studied cases addressing misinformation on the recurring narrative related to vaccines not working or not being sufficiently tested in the two studied types of sources across the different phases of the pandemic

**Figure 17**. Percentage of studied cases addressing misinformation on the recurring narrative related to lockdown or other measures being used to control the population or limit their freedom in the two studied types of sources across the different phases of the pandemic

**Figure 18**. Percentage of studied cases addressing misinformation on the recurring narrative related to George Soros being involved in some way with the virus, the measures or the vaccines in the two studied types of sources across the different phases of the pandemic

**Figure 19**. Percentage of studied cases addressing misinformation on the recurring narrative related to the connection between 5G and the pandemic in the two studied types of sources across the different phases of the pandemic

**Figure 20**. Percentage of studied cases addressing misinformation on the recurring narrative related to the vaccination process in the two studied types of sources across the different phases of the pandemic

**Figure 21**. Percentage of studied cases addressing misinformation on the recurring narrative related to testing in the two studied types of sources across the different phases of the pandemic

**Figure 22**. Percentage of studied cases addressing misinformation on the recurring narrative related to the implementation of measures in the two studied types of sources across the different phases of the pandemic

**Figure 23**. Percentage of studied cases addressing misinformation on the recurring narrative related to the activity of public health authorities in the two studied types of sources across the different phases of the pandemic

**Figure 24**. Percentage of studied cases addressing misinformation on the recurring narrative related to warnings about phishing and other cybercrimes in the two studied types of sources across the different phases of the pandemic
